# BmATG5, BmATG6 and BmATG8 Are Involved in Autophagy and Apoptosis During Metamorphosis Induced by Cadmium in *Bombyx mori*

**DOI:** 10.3390/ijms27094036

**Published:** 2026-04-30

**Authors:** Cuijie Cui, Meihereayi Mutailifu, Maierhaba Sailaijiang, Xutong Wang, Yuning Zhang, Danni Chen, Kun Xie

**Affiliations:** 1 Key Laboratory of Biological Resources and Ecology of Pamirs Plateau in Xinjiang Uygur Autonomous Region, College of Life and Geographic Sciences, Kashi University, Kashi 844000, China; ccj19987654321@163.com (C.C.); miray1201@outlook.com (M.M.); marhaba0712@163.com (M.S.); wangxutong2002@163.com (X.W.); zyn20021218@163.com (Y.Z.); 13267822589@163.com (D.C.); 2 College of Biological and Agricultural Sciences, Honghe University, Mengzi 661199, China

**Keywords:** cadmium, autophagy, apoptosis, BmATG5, BmATG6, BmATG8, *Bombyx mori*, heavy metal toxicity

## Abstract

Cadmium (Cd) is a pervasive environmental contaminant with potent cytotoxic effects in a wide range of organisms. Although autophagy and apoptosis are recognized as major cellular responses to heavy metal stress, the molecular basis of Cd-induced cell death in insects remains insufficiently understood. In this study, we used fifth-instar day-4 (5L4D) larvae of Bombyx mori and the silkworm-derived Bm-12 cell line to investigate the involvement of three core autophagy-related proteins, *Bombyx mori* Autophagy-related protein 5(BmATG5), *Bombyx mori* Autophagy-related protein 6(BmATG6), and Autophagy-related protein 8(BmATG8), in Cd-induced autophagy and apoptosis. Exposure to CdCl_2_ markedly induced autophagic and apoptotic responses in both larval midgut tissue and Bm-12 cells, as demonstrated by monodansylcadaverine(MDC) staining, Lyso-Tracker Red staining, DAPI and Hoechst 33258 staining, and DNA fragmentation assays. qPCR and Western blot analyses showed significant upregulation of BmATG5, BmATG6, and BmATG8 following Cd exposure. Notably, the cleaved forms tBmATG5-N (24 kDa) and tBmATG6-C (35 and 37 kDa), as well as the lipidated form BmATG8-PE (12 kDa), accumulated substantially under Cd stress. In parallel, intracellular Ca^2+^ levels and calpain activity were significantly increased, suggesting activation of a calcium-dependent regulatory pathway. Pharmacological inhibition experiments further indicated that autophagy and apoptosis are functionally interconnected during the Cd response. Collectively, these findings demonstrate that BmATG5, BmATG6, and BmATG8, together with their processed forms, play central roles in coordinating autophagy–apoptosis crosstalk during Cd-induced cytotoxicity in *Bombyx mori*. This study provides new mechanistic insight into heavy metal toxicity in insects and expands our understanding of stress-induced programmed cell death during silkworm metamorphosis.

## 1. Introduction

The silkworm, *Bombyx mori*, is not only an economically important insect in sericulture but also a well-established lepidopteran model for studies of development, endocrinology, physiology, and environmental toxicology [1]. Owing to its well-characterized life cycle and experimental tractability, *Bombyx mori* provides an excellent system for investigating how environmental stressors affect insect growth and tissue homeostasis. Among such stressors, heavy metal pollution has emerged as a major ecological concern accompanying industrialization and intensive agricultural activity. Cadmium (Cd), in particular, is one of the most toxic heavy metals because of its high environmental persistence, strong bioaccumulation potential, and broad-spectrum biological toxicity [2].

In sericultural environments, Cd can enter the food chain through contaminated soil and irrigation water, ultimately accumulating in mulberry leaves. Silkworms feeding on contaminated leaves are therefore at risk of chronic Cd exposure, which may impair larval development, metamorphosis, and silk production. The midgut is especially vulnerable in this context, as it serves as the primary site for digestion, nutrient absorption, and detoxification while simultaneously acting as the first barrier against ingested toxicants [3]. At the cellular level, Cd is known to disrupt redox balance, damage DNA, perturb mitochondrial and endoplasmic reticulum function, and alter calcium homeostasis, thereby triggering a range of stress responses that culminate in programmed cell death [4].

Among these responses, autophagy and apoptosis are of particular interest. Both are highly conserved processes that play essential roles in insect metamorphosis, especially during the degeneration and remodeling of larval tissues such as the midgut, fat body, and silk gland [5]. Increasing evidence indicates that autophagy and apoptosis do not act in isolation; rather, they are tightly interconnected and can either cooperate or antagonize each other depending on the cellular context and intensity of the stimulus [6]. This interplay is orchestrated by a set of autophagy-related proteins (ATGs), among which ATG5, ATG6, and ATG8 occupy central regulatory positions. ATG5 is required for autophagosome elongation, ATG6/Beclin-1 is involved in autophagy initiation, and ATG8 is essential for autophagosome membrane expansion and is widely used as a molecular marker of autophagy [7].

Beyond their canonical roles in autophagy, ATG5 and ATG6 are also implicated in apoptosis. In mammalian cells, calpain-mediated cleavage of ATG5 yields a truncated fragment that translocates to mitochondria and promotes apoptotic signaling [8]. Likewise, Beclin-1 can be cleaved by caspases, generating C-terminal fragments that suppress autophagy and enhance apoptosis [9]. These findings suggest that proteolytic processing of ATG proteins may represent a conserved molecular mechanism linking autophagy to apoptosis under stress conditions. Whether similar regulatory events occur in insects in response to heavy metal exposure, however, remains largely unknown.

In insects, several studies have documented the extensive activation of autophagy and apoptosis during metamorphic tissue degeneration. In Drosophila and *Bombyx mori*, peaks in 20-hydroxyecdysone (20E) signaling are associated with robust autophagic activity in the fat body and midgut, often accompanied by caspase activation and apoptotic morphology [10,11]. Franzetti et al. further showed that autophagy precedes apoptosis in degenerating larval midgut and silk gland tissues during silkworm metamorphosis [12]. In our previous work, we demonstrated that starvation or hormonal stimulation induces autophagy-associated apoptosis in Bm-12 cells, and that BmATG5 and BmATG6 participate in these processes [13,14]. Despite these advances, the role of ATG proteins in heavy metal-induced autophagy–apoptosis crosstalk in silkworms has not been systematically investigated.

Although Cd-induced autophagy and apoptosis have been extensively studied in mammalian systems [15,16,17], comparable studies in insects remain limited. In particular, the contribution of core autophagy regulators to Cd-induced cytotoxicity in silkworm midgut tissue has not been clearly defined. In the present study, we therefore used 5L4D silkworm larvae and the Bm-12 cell line as in vivo and in vitro models to examine the roles of BmATG5, BmATG6, and BmATG8 in Cd-induced autophagy and apoptosis. We further assessed the accumulation of their processed forms and explored the possible involvement of Ca^2+^/calpain signaling. Our results provide evidence that these ATG proteins function at the interface between autophagy and apoptosis during Cd stress and offer new insight into the molecular toxicology of heavy metals in insects.

## 2. Results

### 2.1. Cd Stress Induces Bm-12 Cell Death and Elevated ROS Levels

**Figure 1:**
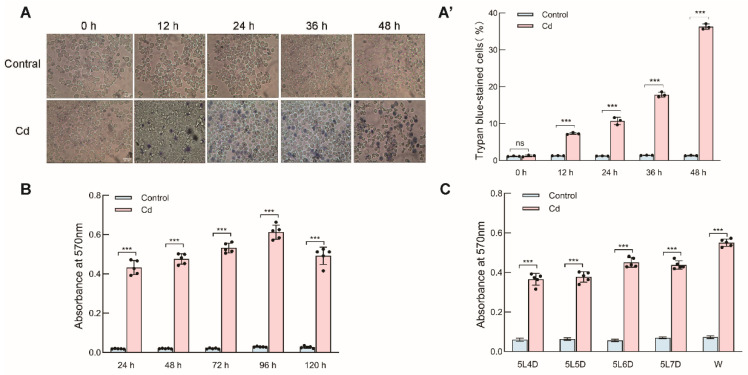
Cell death induced by CdCl_2_ in Bm-12 cells and ROS level of midgut and Bm-12 cells. Cell death was detected by trypan blue staining ((**A**) Scale bar, 20 μm) and dead cells were counted (**A′**). ROS level was detected by spectrophotometry in the midgut of *Bombyx mori* and Bm-12 cells (**B**,**C**). These data are presented as the mean ± SD (*n* = 3) and were analyzed by one-way ANOVA with Sidak’s multiple comparisons test. Exact *p*-values are reported in the text. *** means *p* < 0.001 (highly significant), while ns means not significant (*p* ≥ 0.05).

To assess the cytotoxicity of Cd in silkworm cells, Bm-12 cells were exposed to CdCl_2_ for up to 48 h. Trypan blue staining revealed a clear time-dependent increase in cell death following Cd treatment. Dead cells were already detectable at 12 h, accounting for 8.78% of the total population, and this proportion increased progressively to 36.29% at 48 h, significantly exceeding that in the untreated controls (Figure 1A,A′). Because Cd-induced toxicity is often associated with oxidative stress, we next measured the ROS levels in both silkworm midgut tissue and Bm-12 cells. In both systems, Cd treatment caused a marked increase in ROS accumulation relative to the corresponding controls (Figure 1B,C). These results indicate that Cd provokes oxidative damage and compromises cell viability in silkworm tissues and cultured cells.

### 2.2. Cd Stress Induces Autophagy in Bm-12 Cells

**Figure 2:**
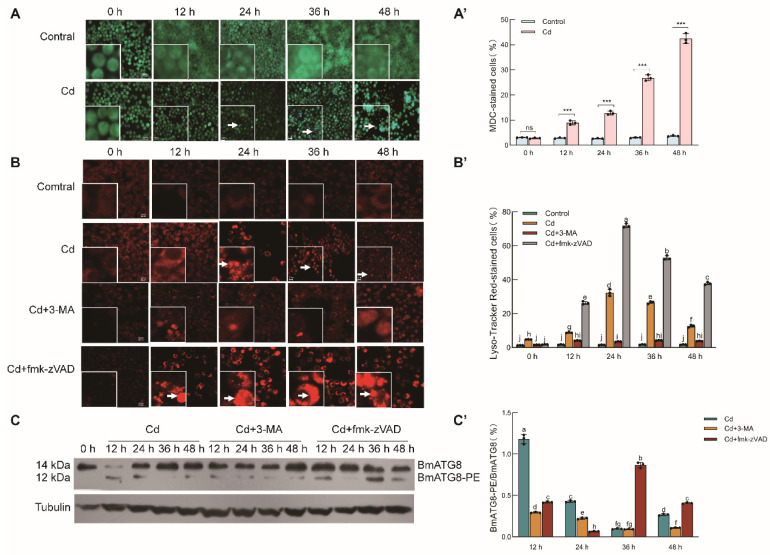
Autophagic response of Bm-12 cells to cadmium stress. Bm-12 cells were treated with 2 µM CdCl_2_. Representative images of MDC-stained Bm-12 cells obtained by laser scanning confocal microscopy (Zeiss LSM 510, Oberkochen, Germany) (**A**) Scale bar, 20 μm). The arrows denote autophagosomes or autolysosomes Quantitative analysis of autolysosomes (**A′**). Cells were further treated with Cd alone, Cd + 3-MA, or Cd + z-VAD-fmk. Lyso-Tracker Red staining showing autolysosome numbers ((**B**,**B′**) scale bar, 20 μm), The arrows denote autolysosome. Expression level of BmATG8-PE detected by Western blotting in different treatment groups (**C**), with β-actin as the loading control. Densitometric analysis of BmATG8-PE levels (**C′**). These data are presented as the mean ± SD (*n* = 3) and were analyzed by two-way ANOVA with Sidak’s multiple comparisons test. *** means *p* < 0.001 (highly significant), ns means not significant.

To determine whether Cd activates autophagy in Bm-12 cells, we evaluated autolysosome formation by MDC and Lyso-Tracker Red staining. Autophagic structures became apparent as early as 12 h after Cd exposure (Figure 2A,B), and quantitative analysis showed that 10.79% of cells were MDC-positive at this time point (Figure 2A′). Co-treatment with the autophagy inhibitor 3-MA markedly reduced autolysosome formation, confirming that the observed vesicular structures reflected autophagic activity. In contrast, co-treatment with the pan-caspase inhibitor Z-VAD-fmk led to an increase in autolysosome accumulation, with a peak at 24 h (Figure 2B′), suggesting that suppression of apoptosis enhances the autophagic response under Cd stress.

To further substantiate these observations, we examined the autophagy marker BmATG8. Western blot analysis showed that the lipidated form, BmATG8-PE, became detectable after 12 h of Cd treatment (Figure 2C) and accumulated over time. Consistent with the staining results, BmATG8-PE levels were reduced in the Cd + 3-MA group but were highest in the Cd + Z-VAD-fmk group, particularly at 36 h (Figure 2C′). Together, these data demonstrate that Cd induces a robust autophagic response in Bm-12 cells and further suggest reciprocal regulation between autophagy and apoptosis during Cd exposure.

### 2.3. Cd Stress Induces Autophagy in Silkworm Midgut Tissues

**Figure 3:**
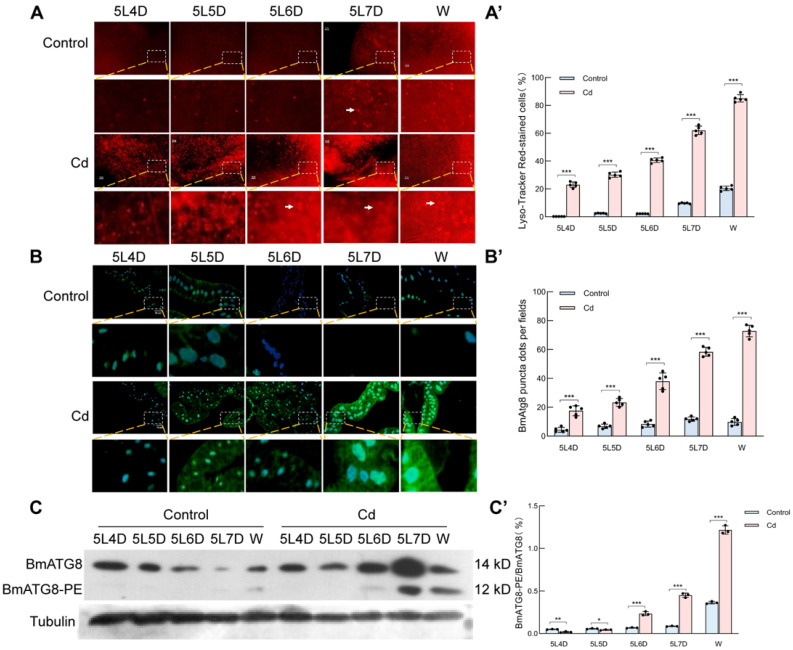
Autophagic response in the midgut tissue of silkworms under cadmium stress. Representative images of midgut tissue sections stained with Lyso-Tracker Red. (**A**) Scale bar, 20 μm) The arrows denote autolysosome. Quantification of autolysosomes from the Lyso-Tracker Red staining (**A**′). Immunohistochemical detection of BmATG8-PE using an FITC-labeled anti-BmATG8 polyclonal antibody ((**B**) scale bar, 20 μm). Quantification of BmATG8-PE fluorescent puncta. (**B′**) Expression of ATG8-PE in the midgut tissue under cadmium stress, as detected by Western blotting (**C**). Densitometric analysis of ATG8-PE levels (**C′**). These data are presented as the mean ± SD (*n* = 3) and were analyzed by one-way ANOVA. * means *p* < 0.05 (statistically significant), ** means *p* < 0.01 (very significant), *** means *p* < 0.001 (highly significant).

We next asked whether Cd also activates autophagy in vivo. Silkworm larvae were fed Cd-treated mulberry leaves from 5L3D to the wandering stage, and midgut tissues were collected for analysis. Lyso-Tracker Red staining revealed a pronounced increase in acidic autolysosomal structures in the midgut of Cd-treated larvae compared with the controls (Figure 3A,A′). Consistently, immunohistochemical staining with anti-BmATG8 antibody showed enhanced fluorescence in Cd-exposed midgut tissue, with the strongest signals observed at 5L7D and the wandering stage (Figure 3B,B′). Western blot analysis further confirmed that BmATG8-PE became detectable from 5L6D onward and increased progressively during subsequent stages under Cd treatment (Figure 3C,C′). These findings indicate that Cd exposure markedly stimulates autophagy in silkworm midgut tissue during late larval development.

### 2.4. Cd Stress Induces Apoptosis in Silkworm Midgut Tissues and Bm-12 Cells

**Figure 4:**
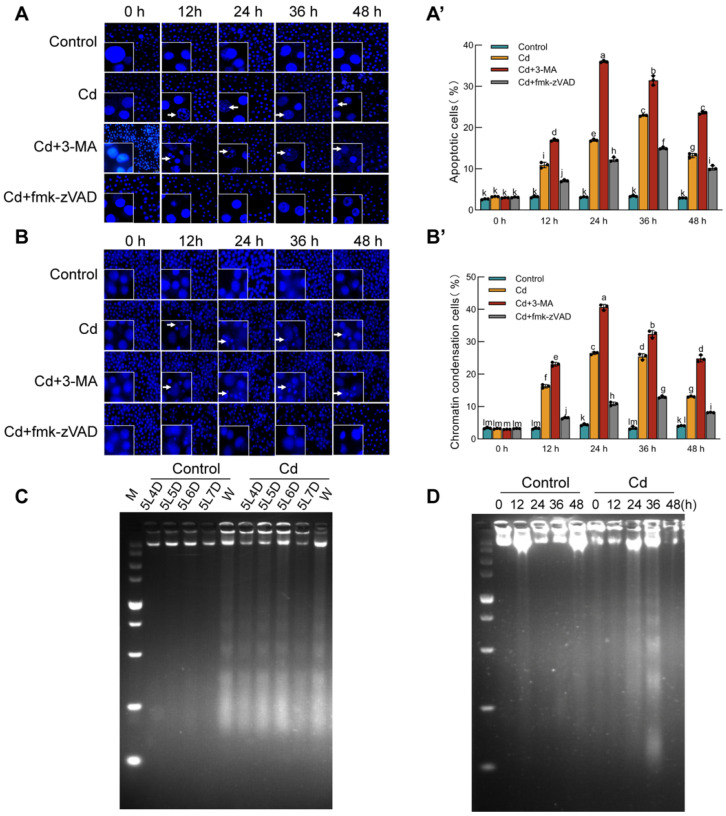
Apoptotic response of the silkworm midgut tissue and Bm-12 cells to cadmium stress. Bm-12 cells from the cadmium chloride treatment group, Cd + 3-MA group, and Cd + z-VAD-fmk group were stained with Hoechst 33258, and observed under a laser scanning confocal microscope ((**A**,**B**) scale bar, 20 μm). The arrows denote apoptotic body. Quantitative analysis of apoptotic bodies (**A′**,**B′**). These data are presented as the mean ± SD (*n* = 3) and were analyzed by two-way ANOVA with Sidak’s multiple comparisons test. Nuclear DNA was extracted from the silkworm midgut tissue and Bm-12 cells and analyzed by agarose gel electrophoresis to detect the DNA fragment (**C**,**D**).

Because autophagy and apoptosis frequently occur in parallel under cellular stress, we examined whether Cd also promotes apoptotic cell death. DAPI and Hoechst 33258 staining revealed typical apoptotic features in Bm-12 cells, including chromatin condensation and apoptotic body formation, beginning at 12 h after Cd exposure (Figure 4A,B). Notably, the number of apoptotic bodies was greatest in the Cd + 3-MA group at 36 h (Figure 4A′,B′), indicating that the inhibition of autophagy potentiates apoptosis under Cd stress. DNA ladder assays further showed characteristic internucleosomal DNA fragmentation in both the Cd-treated Bm-12 cells and silkworm midgut tissue (Figure 4C,D). Collectively, these data demonstrate that Cd induces apoptosis in both the in vitro and in vivo silkworm models.

### 2.5. Cd Stress Elevates Ca^2+^ Concentration and Calpain Activity in Bm-12 Cells

**Figure 5:**
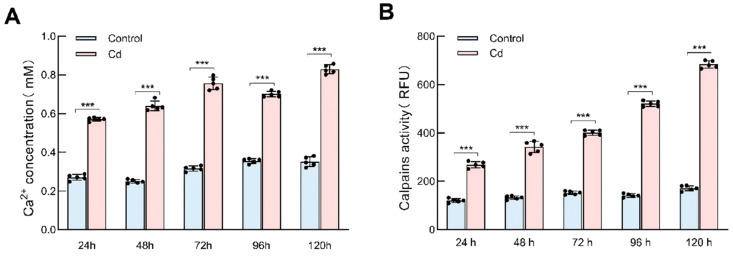
Detection of intracellular Ca^2+^ concentration and calpain activity in cadmium-stressed silkworm Bm-12 cells. Intracellular Ca^2+^ concentration (**A**) and calpain activity (**B**) in Bm-12 cells were measured using a Calcium Assay Kit (Abcam, ab102505) and a Calpain Activity Assay Kit (Abcam, ab65308), respectively. Fluorescence/absorbance was measured at 575 nm for Ca^2+^ and 400 nm for calpain. The data are presented as the mean ± SD (*n* = 3) and were analyzed by two-way ANOVA with Sidak’s multiple comparisons test. *** means *p* < 0.001 (highly significant).

To investigate the upstream signaling events involved in Cd-induced autophagy and apoptosis, we measured the intracellular Ca^2+^ levels and calpain activity in Bm-12 cells after Cd exposure. Both parameters increased progressively over time and were significantly higher in the Cd-treated cells than in the controls (Figure 5A,B). Given that calpain is a Ca^2+^-dependent protease capable of cleaving key autophagy regulators, these findings suggest that Cd-induced calcium dysregulation may contribute to proteolytic remodeling of the autophagic machinery and thereby facilitate the transition toward apoptosis.

### 2.6. Cadmium Promotes Expression and Processing of BmATG5, BmATG6, and BmATG8

**Figure 6:**
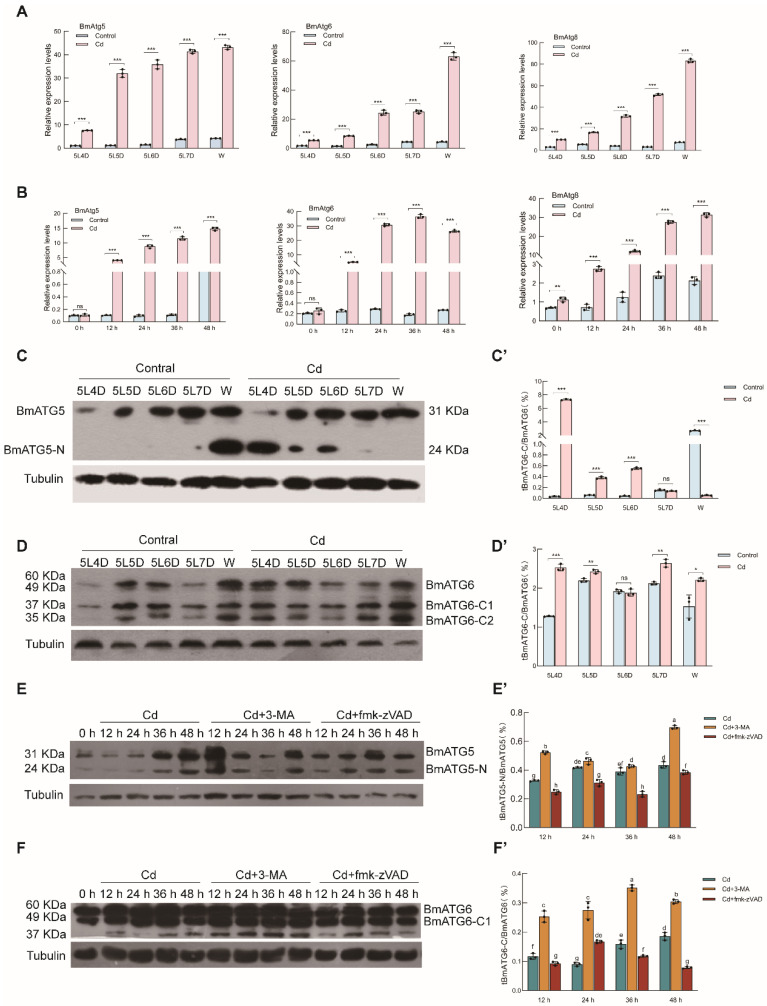
Response of BmATG5, BmATG6, and their truncated fragments to cadmium-induced autophagy and apoptosis. The transcriptional levels of the autophagy-related genes BmATG5, BmATG6, and BmATG8 in the midgut tissue and Bm-12 cells were detected by qPCR (**A**,**B**). The expression of BmATG5, BmATG6, and their truncated fragments—tBmATG5-N (24 kDa) and tBmATG6-C (35 kDa and 37 kDa)—in cadmium-induced midgut tissue and Bm-12 cells was examined by Western blotting (**C**–**F**), with β-actin as the loading control. Densitometric scanning analysis of the truncated fragments is presented in panels (**C′**–**F′**). These data are presented as the mean ± SD (*n* = 3) and were analyzed by one-way ANOVA with Sidak’s multiple comparisons test. * means *p* < 0.05 (statistically significant), ** means *p* < 0.01 (very significant), *** means *p* < 0.001 (highly significant), ns means not significant.

To further define the molecular response to Cd, we examined the expression of BmATG5, BmATG6, and BmATG8 at both the mRNA and protein levels. qPCR analysis showed that all three genes were significantly upregulated in Cd-treated midgut tissue and Bm-12 cells relative to the controls (Figure 6A,B). Western blotting revealed not only increased expression of the full-length proteins but also a marked accumulation of the truncated BmATG5 fragment tBmATG5-N (24 kDa), two C-terminal BmATG6 fragments, tBmATG6-C1 and tBmATG6-C2 (35 and 37 kDa), and the lipidated form BmATG8-PE (12 kDa) (Figure 6C–F′). Strikingly, the truncated forms of BmATG5 and BmATG6 were most abundant in the Cd + 3-MA group, in which apoptosis was also most pronounced. These observations suggest that autophagy inhibition favors proteolytic processing of BmATG5 and BmATG6 and promotes apoptotic progression. Overall, the data support a model in which BmATG5, BmATG6, and BmATG8 act as central regulators of autophagy–apoptosis crosstalk during Cd-induced stress in *Bombyx mori*.

## 3. Discussion

### 3.1. Cd Induces Autophagy and Apoptosis in Bombyx mori

In the present study, we demonstrated that cadmium exposure elicits both autophagy and apoptosis in silkworm midgut tissue and Bm-12 cells [18], and that this response is closely associated with the coordinated regulation of BmATG5, BmATG6, and BmATG8. Our findings provide molecular evidence that these core autophagy-related proteins participate not only in autophagic activation but also in the shift from a cytoprotective response toward apoptotic cell death under heavy metal stress [19,20,21,22].

Cd is a non-essential and highly toxic metal that disrupts multiple aspects of cellular homeostasis. Although it does not directly generate free radicals through redox cycling, Cd is well-known to promote oxidative stress indirectly by impairing antioxidant defenses, interfering with mitochondrial function, and provoking ROS accumulation [23,24]. In agreement with this established mode of action, we observed a significant increase in ROS levels in both the Cd-treated midgut tissue and Bm-12 cells. Because ROS can act as upstream signals for both autophagy and apoptosis, the elevated oxidative stress detected here is likely to represent an early trigger of the downstream responses observed in our study.

Autophagy is generally regarded as an adaptive mechanism that promotes cell survival under stress by removing damaged organelles and misfolded proteins. However, sustained or excessive autophagy can also accompany, or even facilitate, cell death [25]. In the present work, multiple lines of evidence—including MDC staining, Lyso-Tracker Red staining, BmATG8 immunolocalization, and increased BmATG8-PE levels—clearly demonstrate that Cd activates autophagy in both cultured cells and larval midgut tissue. The fact that Z-VAD-fmk further increased autolysosome accumulation and the BmATG8-PE levels suggests that when apoptosis is blocked, cells may rely more heavily on autophagy as a compensatory or protective mechanism. Conversely, 3-MA-mediated inhibition of autophagy enhanced apoptotic morphology and DNA fragmentation, indicating that autophagy initially plays a cytoprotective role during Cd stress in *Bombyx mori*.

At the same time, our data show that Cd exposure robustly induces apoptosis, as evidenced by chromatin condensation, apoptotic body formation, and DNA laddering. Importantly, apoptosis was more pronounced when autophagy was inhibited, reinforcing the idea that the two pathways are functionally interconnected. This reciprocal relationship is consistent with previous reports in insect and mammalian systems, where autophagy can transiently delay apoptosis under moderate stress but eventually gives way to cell death when the damage becomes irreversible [26,27]. The silkworm midgut, which naturally undergoes extensive remodeling during metamorphosis, may be especially sensitive to such a transition.

### 3.2. Cleavage of BmATG5 and BmATG6 Correlates with Apoptosis

One of the most significant findings of this study is the accumulation of truncated forms of BmATG5 and BmATG6 under Cd stress. In mammalian cells, ATG5 cleavage by calpain generates a pro-apoptotic fragment that translocates to mitochondria and promotes cytochrome c release [8,28]. Our observation that intracellular Ca^2+^ levels and calpain activity increase progressively after Cd treatment strongly supports the idea that a similar calcium-dependent cleavage mechanism operates in silkworm cells. The marked accumulation of tBmATG5-N, particularly under conditions where autophagy was inhibited and apoptosis was enhanced, suggests that BmATG5 may function as a molecular switch that links autophagic activation to apoptotic execution.

BmATG6 appears to play a similarly important role. As the insect homolog of Beclin-1, BmATG6 is expected to participate in the initiation of autophagy. However, we detected two C-terminal cleavage products of approximately 35 and 37 kDa that accumulated under Cd treatment. In mammalian systems, caspase-mediated Beclin-1 cleavage suppresses autophagy and promotes apoptosis by generating fragments with pro-death activity [9]. Although the protease responsible for BmATG6 cleavage remains to be identified, the presence of these fragments under Cd stress, especially when apoptosis is prominent, suggests that proteolytic processing of BmATG6 may contribute to the conversion of autophagy from a survival pathway into an apoptotic one in *Bombyx mori*.

In contrast, BmATG8 behaved primarily as a marker and effector of autophagic activation. The progressive increase in BmATG8-PE in both the midgut tissue and Bm-12 cells confirms that Cd stimulates autophagosome formation and/or autophagic flux. Because ATG8 lipidation is essential for autophagosome biogenesis, the induction of BmATG8-PE likely reflects the activation of the core autophagy machinery in response to Cd-induced cellular injury. The enhanced BmATG8-PE signal observed after Z-VAD-fmk treatment further emphasizes the dynamic balance between autophagy and apoptosis and suggests that inhibition of one pathway can alter the intensity of the other.

The in vivo component of this study is particularly relevant to environmental toxicology. The silkworm midgut is directly exposed to dietary contaminants and therefore represents a physiologically meaningful target tissue for investigating the effects of heavy metals. Our data indicate that Cd not only activates stress-responsive pathways at the cellular level but may also interfere with normal tissue remodeling during metamorphosis. Because the degeneration of larval midgut tissue is a tightly regulated developmental event, exogenous Cd exposure may distort this process by prematurely or excessively activating autophagy and apoptosis. Such disturbances could ultimately compromise larval fitness, metamorphic success, and silk production.

### 3.3. Ca^2+^/Calpain Hypothesis and Limitations

**Figure 7:**
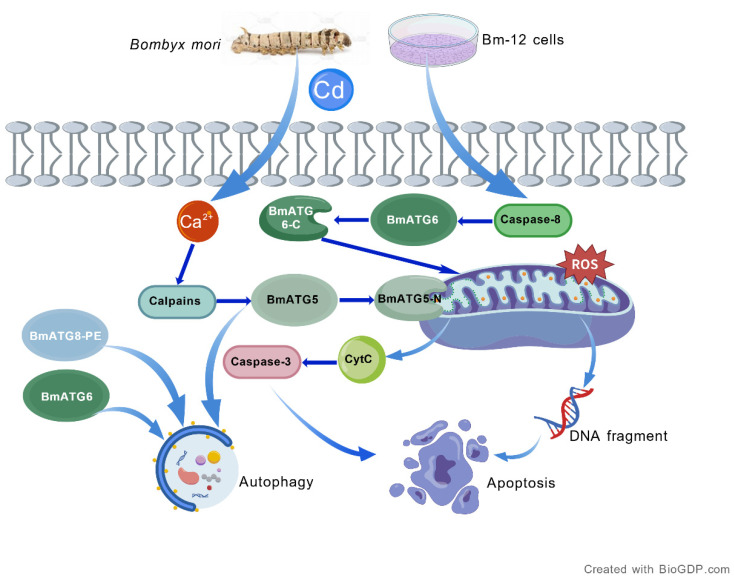
Molecular mechanism of autophagy and apoptosis induced by the heavy metal cadmium in *Bombyx mori.* Cadmium triggers an increase in intracellular calcium ions and caspase-8 levels in Bm-12 cells. On the one hand, elevated calcium ions activate calpains, which in turn cleave BmATG5 into BmtATG5-N that localizes to the mitochondrial membrane. On the other hand, activated caspase-8 cleaves BmATG6 into BmtATG6-C, which also translocates to the mitochondrial membrane. These events collectively promote the activation of ROS, formation of DNA ladders, and release of cytochrome c (CytC) from mitochondria. Subsequently, CytC further activates caspase-3, thereby inducing caspase-dependent apoptosis. The proposed calpain-mediated cleavage events in this model are based on correlative data and remain hypothetical until validated by calpain-specific inhibition or genetic approaches.

Taken together, our results support a model in which Cd exposure first induces oxidative stress and calcium imbalance, leading to autophagic activation through the increased expression of BmATG5, BmATG6, and BmATG8. With sustained stress, elevated intracellular Ca^2+^ and calpain activity promote the proteolytic processing of BmATG5 and possibly BmATG6, thereby shifting the balance from autophagy toward apoptosis. In this framework, BmATG5 and BmATG6 serve as dual-function regulators at the intersection of the two pathways [28], whereas BmATG8 reflects the activation status of the autophagic machinery. This mechanistic model provides a new perspective on heavy metal-induced programmed cell death in insects (Figure 7).

Despite these advances, several limitations should be acknowledged. First, although pharmacological inhibitors were informative, genetic approaches such as RNA interference or CRISPR-mediated gene disruption would provide stronger evidence for the causal roles of BmATG5, BmATG6, and BmATG8. Second, the identity of the proteases responsible for BmATG6 cleavage remains to be clarified. Third, MDC and Lyso-Tracker staining primarily detect acidic vesicular compartments and do not distinguish between increased autophagic flux and impaired lysosomal degradation. Future studies should employ flux assays (e.g., bafilomycin A1) and tandem fluorescent reporters (e.g., mCherry-GFP-LC3) to quantify autophagic flux directly. Fourth, while DNA laddering and nuclear morphology are established indicators of apoptosis, direct measurement of caspase-3-like activity or cleaved caspase-3 by Western blot would strengthen the apoptosis conclusion and will be performed in future studies. Finally, our data do not prove a causal role for Ca^2+^/calpain in ATG processing; this remains a hypothesis to be tested. Addressing these questions will help refine the molecular model proposed here.

Importantly, while our pharmacological and correlative data support the involvement of BmATG5, BmATG6, and BmATG8 in Cd-induced autophagy and apoptosis, we have not yet performed direct loss-of-function experiments (e.g., RNAi-mediated knockdown of individual atg genes). Such genetic perturbations will be essential in future studies to establish causal relationships between specific ATG protein processing and apoptotic outcomes.

It should be emphasized that although the correlation between elevated calpain activity and ATG fragment accumulation is strong, direct evidence for calpain-mediated cleavage of BmATG5 and BmATG6 is still lacking. Calpain inhibitor experiments will be required to test this hypothesis in future studies.

## 4. Materials and Methods

### 4.1. Insects and Cell Culture

Silkworms (*Bombyx mori*, *Qingsong × Haoyue*) were reared on fresh mulberry leaves under standard laboratory conditions at 25 °C and 70% relative humidity. Larvae at the fifth instar, day 4 (5L4D), were used for all in vivo experiments. The silkworm-derived ovarian cell line Bm-12 was kindly provided by Professor Chuanxi Zhang (Zhejiang University, Hangzhou, China) and maintained in TNM-FH medium (Hyclone, AXL50928, Logan, UT, USA) supplemented with 10% fetal bovine serum.

### 4.2. Cadmium Treatment of Silkworm Larvae

Larvae were fed fresh mulberry leaves until the fifth instar, day 3 (5L3D), and then randomly assigned to control and Cd treatment groups (20 larvae per group). For Cd exposure, mulberry leaves were soaked in 80 mg/L CdCl_2_ solution and fed continuously until the wandering stage (W). Control larvae received untreated mulberry leaves over the same period. Midgut tissues were collected at successive developmental stages from 5L4D to W, immediately frozen in liquid nitrogen, and stored for subsequent analyses.

### 4.3. Cell Treatment

Exponentially growing Bm-12 cells were assigned to the following groups: control, Cd, Cd + 3-MA, and Cd + Z-VAD-fmk. Cells in the Cd group were exposed to 2 µM CdCl_2_. For inhibitor treatments, cells were co-incubated with CdCl_2_ (2 µM) and either 3-methyladenine (3-MA, 10 µM; Sigma-Aldrich, St. Louis, Missouri, USA) or Z-VAD-fmk (20 µM; Sigma-Aldrich, USA). Culture medium containing the corresponding reagents was refreshed every 24 h for 4 consecutive days. Cells were harvested at 0, 12, 24, 36, and 48 h for subsequent experiments.

### 4.4. Trypan Blue Staining

Bm-12 cells grown on coverslips were treated with 2 µM CdCl_2_ and stained with 0.4% trypan blue for 1 min after removal of the culture medium. Cells were then washed twice with phosphate-buffered saline (PBS), mounted on glass slides, and examined using a Zeiss LSM510 (Oberkochen, Germany) laser scanning confocal microscope. The percentage of dead cells (trypan blue positive) was calculated by counting at least 200 cells per coverslip in five random fields of view.

### 4.5. Detection of Reactive Oxygen Species

Reactive oxygen species (ROS) levels in silkworm hemolymph and Bm-12 cells were determined using the nitroblue tetrazolium (NBT) reduction assay as previously described by Yang et al. [29].

### 4.6. MDC Staining

To detect autophagic vacuoles, collected Bm-12 cells were washed with PBS (0.1 mol/L, pH 7.0) and incubated with monodansylcadaverine (MDC; Sigma-Aldrich, USA) at a final concentration of 50 µg/mL for 30 min at room temperature in the dark. After washing twice with PBS, cells were mounted on slides and observed under a Zeiss LSM510 confocal microscope. At least 200 cells per group were counted in five random fields.

### 4.7. Lyso-Tracker Red Staining

Midgut tissues and Bm-12 cells were washed with PBS, fixed in 3.7% formaldehyde for 5 min, and stained with Lyso-Tracker Red (Thermo Fisher Scientific, Waltham, MA, USA) at a final concentration of 50 nM for 1 h at room temperature in the dark. Fluorescence was visualized using confocal microscopy. At least 200 cells per group were counted in five random fields.

### 4.8. DAPI Staining

Bm-12 cells were washed with PBS, fixed in 3.7% formaldehyde for 5 min, dehydrated through a graded ethanol series (30%, 50%, 70%, 90%, and 100%), and stained with DAPI (1 µg/mL) for 30 min at room temperature in the dark. Nuclear morphology was examined by confocal microscopy.

### 4.9. Hoechst 33258 Staining

Bm-12 cells were washed with PBS and stained with Hoechst 33258 solution (10 µg/mL final concentration; Beyotime, Shanghai, China) for 5 min at 37 °C. After two PBS washes, cells were examined under a confocal microscope. At least 200 cells per group were counted in five random fields.

### 4.10. DNA Ladder Assay

Collected cells were centrifuged at 2000 rpm for 5 min at 4 °C, and pellets were lysed in buffer containing 20 mM EDTA, 100 mM Tris-HCl (pH 8.0), and 0.8% SDS. Samples were then incubated with RNase (10 mg/mL; TIANGEN, M1105, Beijing, China) at 37 °C for 1 h and with proteinase K (20 mg/mL; TIANGEN, K0012, China) at 55 °C overnight. Extracted DNA was separated on 2.5% agarose gels to assess apoptotic DNA fragmentation.

### 4.11. Immunohistochemistry

Immunohistochemical analysis of silkworm midgut tissue was performed largely as described by Franzetti et al. [12]. Paraffin-embedded tissue sections were incubated with a self-prepared polyclonal anti-BmATG8 antibody (1:100), followed by FITC-conjugated goat anti-mouse IgG (Beyotime, China). Fluorescence signals were observed under a Zeiss LSM510 confocal microscope.

### 4.12. Measurement of Intracellular Ca^2+^ and Calpain Activity

Intracellular Ca^2+^ concentration and calpain activity in Bm-12 cells were measured using a Ca^2+^ Detection Kit (Abcam, ab102505, Shanghai, China) and a Calpain Activity Assay Kit (Abcam, ab65308), respectively, according to the manufacturers’ instructions. Fluorescence intensity was measured at 575 nm for Ca^2+^ and absorbance at 400 nm for calpain activity.

### 4.13. qPCR Analysis

Total RNA was extracted from midgut tissues and Bm-12 cells, and cDNA was synthesized for quantitative PCR analysis. Relative transcript levels of BmATG5, BmATG6, and BmATG8 were normalized to the internal reference gene and calculated using the 2^−ΔΔCt^ method.

### 4.14. Western Blotting

Total protein was extracted using RIPA lysis buffer containing protease inhibitors. Protein concentration was quantified, and 30 µg protein per lane was separated by SDS-PAGE (12% gel for ATG5/ATG6, 15% gel for ATG8) at 200 V for 45 min. Proteins were transferred to PVDF membranes at 300 mA for 90 min. Membranes were blocked with 5% non-fat milk for 1 h at room temperature, then incubated with self-prepared BmATG5, BmATG6, and BmATG8 polyclonal antibodies (1:1000 in 1% BSA) overnight at 4 °C. Antibody specificity was validated by pre-absorption with corresponding antigen peptides. Goat anti-mouse IgG (Beyotime, 1:5000) was used as the secondary antibody and incubated for 1 h at room temperature. Signals were visualized using Western Lightning ECL reagent(PerkinElmer, Waltham, MA, USA) and ChemiDoc MP imager (Singapore). Densitometric analysis was performed using ImageJ2.0, with β-Tubulin as the loading control for normalization.

### 4.15. Statistical Analysis

All data are presented as the mean ± standard deviation (SD) from three independent experiments. Statistical analyses were performed using GraphPad Prism 9.0 (GraphPad Software, Boston, MA, USA). Differences between two groups were evaluated by the two-tailed Student’s t-test, and multiple-group comparisons were analyzed by one-way ANOVA. Data are presented as the mean ± SD (*n* = 3). * *p* < 0.05, ** *p* < 0.01, *** *p* < 0.001.

## 5. Conclusions

This study demonstrates that cadmium exposure induces both autophagy and apoptosis in silkworm midgut tissues and Bm-12 cells. The autophagy-related proteins BmATG5, BmATG6, and BmATG8, along with their cleavage products (tBmATG5-N, tBmATG6-C, and BmATG8-PE), play crucial roles in mediating Cd-induced cytotoxicity. The elevated intracellular Ca^2+^ concentration and calpain activity following Cd stress contribute to the proteolytic processing of these autophagy proteins, facilitating the transition from autophagy to apoptosis. These findings provide novel molecular insights into the toxicological mechanisms of heavy metals in economically important insects and may inform the development of biomarkers or detoxification strategies for environmental heavy metal pollution. Future studies employing RNA interference or CRISPR-mediated gene disruption will be required to validate the essential roles of these ATG fragments in the autophagy–apoptosis switch.

## Data Availability

The original contributions presented in this study are included in the article. Further inquiries can be directed to the corresponding author.
